# Successful Treatment of Novel H1N1 Influenza related Fulminant Myocarditis with Extracorporeal Life Support

**DOI:** 10.1186/1749-8090-6-164

**Published:** 2011-12-20

**Authors:** Prashant Nanasaheb Mohite, Aron Frederik Popov, Armin Bartsch, Bartlomiej Zych, Dhruva Dhar, Ajay Moza, Heike Krueger, André Ruediger Simon

**Affiliations:** 1 Department of Cardiothoracic Transplantation & Mechanical support, Royal Brompton & Harefield NHS Trust, London, United Kingdom; 2 Department of Thoracic and Cardiovascular Surgery, University of Goettingen, Goettingen, Germany; 3 Department of Critical care & Anaesthesia, Royal Brompton & Harefield Hospitals, London, United Kingdom; 4 Department of Transplant Medicine, Royal Brompton & Harefield Trust, London, United Kingdom

## Abstract

The prevalence of myocardial involvement in influenza infection ranges from 0% to 12%. The 2009 pH1N1 influenza virus, formerly known as swine flu, first appeared in Mexico and the United States of America in March and April 2009 and has swept the globe with unprecedented speed. We report a case of fulminant myocarditis associated with this virus treated successfully using extra-corporal membrane oxygenator.

## Case report

An 18 year old previously fit and well female suffered from lethargy and malaise for six weeks combined with rigors, fever, nausea, vomiting and diarrhea over four days. After collapsing on train, she was admitted to local hospital where she was diagnosed of cardiogenic shock with echocardiogram showing severely impaired left ventricular function with ejection fraction of 20%. She was started on Dobutamine and transferred to our institution for further management. On arrival, the systolic blood pressure was 54 mmHg, sinus tachycardia of 130 per minute, tachypnea and the lactate of 13 mmol/l. The air entry was good and there were no added sounds on auscultation. She arrested shortly after arrival, was intubated and ventilated and after 65 minutes of cardiopulmonary resuscitation (CPR) a Veno - Arterial Extra corporal membrane oxygenator (VA ECMO) was inserted. The decision to put ECMO was based on need of short term circulatory support and emergency situation. The option of short term left ventricular assist device (LVAD) was not feasible as patient was being resuscitated and could not be moved in theatre for LVAD implantation. The left femoral vessels accessed percutaneously by Seldinger technique. A 17 and 21 French cannulae were inserted into Femoral artery and vein respectively and connected to ECMO circuit comprising Levitronic CentriMag pump and Medtronic oxygenator. A 10 French cannula was inserted in Femoral artery for distal limb perfusion and connected to the main arterial cannula by 'Y' connection. Cardiovascular stability could be achieved with initial ECMO flow of 3 l/min and moderate doses of Noradrenalin and Adrenaline targeting a mean arterial pressure of 60 mmHg. Due to the cardiogenic shock and the hypotension caused by the low cardiac output state she developed acute kidney injury and was treated with continuous veno-venous hemofiltration (CVVH). On day 3, she developed compartment syndrome in the leg on the side of ECMO insertion which needed fasciotomy. Viral PCR (Polymerase Chain Reaction) test detected Influenza A RNA in nasal and throat secretions of the patient confirming H1N1 Pandemic strain a week after the admission and she was started on OD 5 mg Oseltamivir. After 10 days of mechanical support, left ventricular function improved significantly and ejection fraction of 50-55% with the ECMO flows turned down to minimal. The ECMO was weaned off but the ischemic left leg continued to deteriorate despite fasciotomy and revision surgery and left above knee amputation had to be performed. Patient was found intact neurologically from the 4^th ^day of ECMO insertion but kept sedated electively till ECMO was out. Weaning from ventilator and CVVH was unremarkable. Follow up investigations with MRI showed an EF 72%, normal volume and thickness of the ventricle and no regional wall motion abnormalities.

## Discussion

The prevalence of myocardial involvement in influenza infection ranges from 0% to 12% [1]. Moreover, 2009 pH1N1 influenza virus (formerly known as swine flu) first appeared in Mexico and the United States in March and April 2009 and has swept the globe with unprecedented speed [2]. Acute myocarditis during influenza infection is a well-known complication, and the clinical expression varies from asymptomatic to fatal congestive cardiac failure and sometimes death [3]. Fulminant myocarditis (FM) is characterized clinically by distinct onset of cardiac symptoms in otherwise young healthy patients after nonspecific flu-like symptoms rapidly resulting in severe ventricular dysfunction and cardiogenic shock. Mortality up to 30% is reported in FM [4]. Influenza-associated fulminant myocarditis in adults is exceedingly rare [5]. Chacko et al in their experience with 2009 pandemic of H1N1 virus reports high incidence of myocardial injury and dysfunction and was associated with high mortality [6]. Few cases of FM secondary to H1N1 influenza infection are reported in the literature [7-10].

Use of mechanical ventricular support device in FM with severe ventricular failure is well established [7,8]. But, the choice of the device is still debated. In the acute forms of myocarditis expected to be on circulatory support for a long time, may be as a bridge to transplant, the implantable LVAD is more appropriate. However, FM is expected to recover in short time and the device is indicated as a bridge-to-recovery. So, an extra-corporal device seems logical [8]. In the present case, the patient was arrested and being resuscitated. In such emergent situations it is difficult to move the patient to theatre and open chest for short term LVAD implantation. Peripheral ECMO insertion is easy, can be done with on-going CPR and at the bed side.

A hospital in New York reports use of a catheter-based mechanical cardiac assist device (Impella 2.5 Cardiac Assist Device, Abiomed, Danvers, MA) in a patient of fulminant myocarditis secondary to H1N1 influenza, but patient could not survive [9]. Komai et al reports unsuccessful use of mechanical circulatory support in FM [10]. However, some recent reports comment about successful outcome in FM following H1N1 infection with the use of mechanical circulatory support in the form of ECMO [11,12]. In the present case, we supported the failing heart in FM due to H1N1 Influenza infection with VA ECMO for 10 days leading to complete recovery. This is the first case of H1N1 related FM in Europe treated successfully with mechanical circulatory support. The myocardial biopsy to prove the origin of myocarditis was not done in view of dilated thinned out ventricular walls; however, typical symptoms and positive viral PCR test for H1N1 were of abundant evidence.

A subset of pH1N1 patients with FM can deteriorate fast. They should be treated in Intensive Therapy Units and monitored carefully for the declining heart function. If not responding to maximum inotropic support, the option of mechanical circulatory support should not be delayed. Peripherally inserted veno-arterial ECMO is efficient short term circulatory support which can be weaned off with recovery of heart function or can be converted to LVAD if respiratory function is optimum and heart do not recover after 3-4 weeks.

## Conclusion

H1N1 Influenza infection can cause Fulminant Myocarditis leading to rapidly progressing heart failure which can be fatal if not treated in time. Early intervention with extra-corporal life support is indicated in fulminant myocarditis associated with H1N1 influenza infection.

## Consent

Written informed consent was obtained from the patient for publication of this case report. A copy of the written consent is available for review by the Editor-in-Chief of this journal.

## Competing interests

The authors declare that they have no competing interests.

## Authors' contributions

PM, AP, BZ, AM, and AS are members of the surgical team. AB was the anaesthetist who was involved in intensive care unit. DD, BZ and HK added important comments. PM and AP wrote the paper. AM and AS co-wrote the manuscript and added important comments to the paper. All authors have read and approved the final manuscript.

## References

[B1] Mamas MA Fraser D Neyses L Cardiovascular manifestations associated with influenza virus infection Int J Cardiol 2008 130 304 309 10.1016/j.ijcard.2008.04.044 18625525

[B2] World Health Organization, Pandemic (H1N1) 2009 http://www.who.int/csr/disease/swineflu/en/index.html

[B3] Potter CW Chronicle of influenza pandemics Nicholson KG Webster R Hay A Textbook of Influenza, Blackwell Science 1997 Oxford, UK 3 18

[B4] Dec GW Jr Palacios IF Fallon JT Aretz HT Mills J Lee DC Johnson RA Active myocarditis in the spectrum of acute dilated ardiomyopathies: clinical features, histologic correlates, and clinical outcome N Engl J Med 1985 312 885 90 10.1056/NEJM198504043121404 3974674

[B5] Ukimura A Izumi T Matsumori A A national survey on myocarditis associated with the 2009 influenza A (H1N1) pandemic in Japan Circ J 2010 74 2193 2199 10.1253/circj.CJ-10-0452 20697177

[B6] Chacko B Peter JV Pichamuthu K Ramakrishna K Moorthy M Karthik R John G Cardiac manifestations in patients with pandemic (H1N1) 2009 virus infection needing intensive care J Crit Care 2011 10.1016/j.jcrc.2011.05.016 21737242

[B7] Wilmot I Morales DL Price JF Rossano JW Kim JJ Decker JA McGarry MC Denfield SW Dreyer WJ Towbin JA Jefferies JL Effectiveness of mechanical circulatory support in children with acute fulminant and persistent myocarditis J Card Fail 2011 17 487 94 10.1016/j.cardfail.2011.02.008 21624737

[B8] Grinda JM Chevalier P D'Attellis N Bricourt MO Berrebi A Guibourt P Fabiani JN Deloche A Fulminant myocarditis in adults and children: bi-ventricular assist device for recovery Eur J Cardiothorac Surg 2004 26 1169 73 10.1016/j.ejcts.2004.05.059 15541979

[B9] Khouzam RN Parizianu C Hafiz AM Chawla S Schwartz R Fulminant myocarditis associated with novel H1N1 influenza A Heart Lung 2011 40 6 566 8 10.1016/j.hrtlng.2011.01.004 21411147

[B10] Komai T Nakazawa G Asai S Ikari Y Fatal fulminant myocarditis associated with novel influenza A (H1N1) infection Eur Heart J 2011 32 283 10.1093/eurheartj/ehq359 20861138

[B11] Liao YC Hsieh YC Chang WC Huang JL Ting CT Wu TJ Fulminant myocarditis in an adult with 2009 pandemic influenza A (H1N1 influenza) infection J Chin Med Assoc 2011 74 130 133 21421208 10.1016/j.jcma.2011.01.028

[B12] Morimoto R Sone T Tsuboi H Mukawa H Morishima I Uesugi M Sasaki H Niwa T Izumi Y Yamamoto T Ichihashi K Kanzaki Y Nagai H Iwata Y A case of fulminant myocarditis associated with novel N1H1 influenza successfully treated by percutaneous cardiopulmonary support system Journal of Cardiology Cases 2010 2 106 110 10.1016/j.jccase.2010.05.004 PMC6265007 30524598

